# Exploring family caregiver challenges in caring for patients with COVID-19 in intensive care units

**DOI:** 10.3389/fpubh.2023.1057396

**Published:** 2023-03-09

**Authors:** Tahereh Najafi Ghezeljeh, Masoud Rezaei, Sahar Keyvanloo Shahrestanaki, Arezoo Sheikh Milani

**Affiliations:** ^1^Nursing and Midwifery Care Research Center, Cardiovascular Nursing Research Center, Rajaie Cardiovascular Medical and Research Center, School of Nursing and Midwifery, Iran University of Medical Sciences, Tehran, Iran; ^2^Nursing Care Research Center, School of Nursing and Midwifery, Iran University of Medical Sciences, Tehran, Iran; ^3^Department of Community Health Nursing, School of Nursing and Midwifery, Shahid Beheshti University of Medical Sciences, Tehran, Iran

**Keywords:** COVID-19, intensive care unit, health engagement, family caregivers, qualitative study

## Abstract

**Background:**

Families of individuals hospitalized in an intensive care unit (ICU) with severe illnesses, such as COVID-19, are experiencing a range of physical and emotional stressors. Identifying the challenges faced by family members and providing support to loved ones battling life-threatening diseases can lead to improved treatment and care for the said family members in a healthcare setting.

**Aim:**

The current study was conducted to explore and understand the experiences of family caregivers caring for their loved ones battling COVID-19 in an ICU.

**Methods:**

This descriptive qualitative study was conducted from January 2021 to February 2022, based on the experiences of 12 family caregivers of patients with COVID-19 hospitalized in the ICU. Data collection was conducted through purposeful sampling using semi-structured interviews. MAXQDA10 software was used for data management, and conventional content analysis was used for qualitative data analysis.

**Results:**

The present study conducted interviews with caregivers to understand their experiences while caring for a loved one in an ICU. Three main themes emerged from the analysis of these interviews: hardship of care trajectory, pre-loss mourning, and contributing factors in resolving family health crises. The first theme, the hardship of care trajectories, encompasses categories such as immersion in the unknown, lack of care facilities, negligence in care, neglect of families by healthcare providers, self-ignorance, and perceived stigma. The second these was pre-loss mourning that included some categories such as emotional and psychological turmoil, witnessing the exhaustion of loved ones, separation suffering, the fearing of loss, anticipatory grief, blame related to the disease causative agents, and perceived helplessness and despair. The third theme was contributing factors in resolving family health crises that included categories of the critical role of family caregivers in health engagement, the role of healthcare professionals in health engagement, and the role of interpersonal factors in health engagement. A total of 80 subcategories were also obtained based on the experiences of the family caregivers.

**Conclusion:**

This study's findings indicate that families can play an important role in resolving their loved ones' health problems in life-threatening situations such as the COVID-19 pandemic. Moreover, healthcare providers must recognize and prioritize family-based care and trust the families' ability to effectively manage health crises. Healthcare providers should also be attentive to the needs of both the patient and their family members.

## Introduction

At the end of December 2019, the Chinese government announced an outbreak of the new Coronavirus, COVID-19, from the Coronavirus family (1, 2). The disease caused by the novel Coronavirus has affected numerous people in many countries (3, 4). The first peak of the outbreak in Iran occurred at the end of March 2019, during which 3,186 new cases were reported every day (5). According to the announcement of the Iranian Ministry of Health, the first confirmed case of COVID-19 was reported on 18 February 2020, and until 22 May 2020, 151,466 people were infected and 7,797 Iranians died (6). School classes were also suspended from 29 February 2020. In an effort to curb the spread of the disease, the government implemented stricter laws since 27 March 2020, including restrictions on entry and exit from certain cities (5). In addition, some people lost some or all of their sources of income during the outbreak. Finally, according to the latest report from the World Health Organization, until 27 May 2022, 7,230,882 people were infected with COVID-19, of whom 141,293 Iranians died (7). Patients diagnosed with COVID-19 require specialized care, particularly in terms of the expertise, knowledge, attitude, and skills of the nursing staff, as well as the availability of necessary equipment and supplies (8). The lack of medical facilities and staff, confusion in the treatment system, the unpredictable nature of the disease, social isolation, and widespread transmission of the virus have had severe consequences for the healthcare systems in more than 200 countries (9). Moreover, the research conducted in Iran shows that families of people infected with COVID-19 and healthcare workers, such as nurses caring for patients with COVID-19, face many challenges. Meanwhile, a lack of care and treatment options, family anxiety and mental health issues, a lack of protective equipment, isolation in personal and family life, and a lack of support all pose challenges (10, 11).

COVID-19 can significantly increase anxiety and even serious mental illnesses among people (12). Recent pandemics, such as the Black Plague, demonstrated that such health crises cause a significant level of fear and anxiety among the population, with a considerable number of individuals becoming fearful of death (13, 14). COVID-19, along with all the changes it has created worldwide, has also affected families (15). As the first and most basic social institution, family plays a significant role in a person's health and his/her ability to adapt to different conditions (16). Many stressful factors, such as the occurrence of an acute and incurable disease or the hospitalization of a family member, can cause the members to experience a wide range of emotions (17–19). Families who are unable to adapt to the situation may experience psychological damage and an emotional crisis (20, 21). Moreover, the hospitalization of a family member in the ICU forces the family to make decisions regarding the treatment plan and care for the patient, which itself can cause confusion (22).

The findings from studies conducted in Iran showed that 68%, 57.3%, and 46.7% of the family members of patients hospitalized in the ICU experienced anxiety, depression, and moderate-to-severe stress, respectively (23). Other studies worldwide also showed that most family members of critically ill patients suffer from anxiety, depression, and post-traumatic stress (24–28). Having a family member battling COVID-19 during the pandemic causes stress for the entire family. Furthermore, should the patient be admitted to the ICU, the psychological toll on the family greatly would have been exacerbated (29–31). In a qualitative study, Bartoli et al. found that the family members of patients hospitalized in the ICU with COVID-19 experienced fear, withdrawal, loneliness, and uncertainty (20).

Family-based care is a popular therapeutic approach that has recently gained popularity. The proponents of this theory believe that, if family members play an active role in their loved ones' mental, physical, and social support, the outcomes of care will be significantly better (29). This type of care has many benefits for family members, such as helping to maintain and continue family relationships and acquiring skills and a sense of competence to care for patients after discharge, as well as many benefits for the patient, such as reducing emotional stress (32).

In addition, each member of the healthcare team should support the family members of patients hospitalized in the ICU (33). Because of their 24-h interaction and close relationships with the patients, ICU nurses are best positioned to meet the needs of patients' families and help them (34).

Critically ill patients with COVID-19 who are admitted to the ICU are among the most critical patients in the healthcare system due to their need for immediate treatment with life-sustaining technology (31, 35). These families experience high levels of emotional distress and often feel a sense of helplessness during the hospitalization of their loved ones. The patient with COVID-19 and his/her hospitalization in the special care department may have a long-term negative psychological impact, including anxiety. It increases the risk of depression, post-traumatic stress disorder, emotional distress, and sleep disorders (31, 36). However, only a few studies have examined the experiences of families whose loved ones were hospitalized in the ICU with COVID-19 (30, 37).

According to the study by Sutton et al. a qualitative method is suitable for identifying and discovering complex human issues and factors that underly them (24). Critical diseases such as COVID-19 are, in relation to personal experiences, multidimensional and influenced by many factors. Given the complex nature of the disease, future investigations in this area would benefit from utilizing a qualitative approach to gain a more in-depth understanding of the experiences and needs of the family caregivers of critically ill patients in the ICU.

The high prevalence of COVID-19 in Iran and the important role of family caregivers in the treatment process highlight the need for qualitative research in this area. Investigations in this field will help the medical team provide the necessary support for the family members of patients with COVID-19 during such a health crisis. The current study was conducted to examine the experiences of family caregivers of patients hospitalized in the ICU with COVID-19 in Iran.

Considering the importance of understanding the experiences of family caregivers with patients with COVID-19, identifying this concept, and identifying the challenges they face, there is a pressing need to understand these challenges to better prepare for future crises. In this regard, qualitative research is the most appropriate method to gain a deep understanding of the challenges faced by family caregivers during the hospitalization of their loved ones with COVID-19 in the ICU. The content analysis approach in this research, based on the necessity that no research has been published to explain the phenomenon in question or the family caregivers' understanding of the existing challenges, seems to be suitable. The findings of the present study can be used in educational, research, and clinical planning to improve services for family caregivers of critically ill patients in the ICU.

## Materials and methods

### Study design

The current study was conducted using the qualitative method of the conventional content analysis approach. This approach is used when the pre-existing theory is limited, and it helps to choose the study's direction and goals, as well as the categories and their names, from the gathered data from the participants (38). This article was prepared based on the Consolidated Criteria for Reporting Qualitative Research (COREQ) guidelines (39, 40) (Supplementary File 1).

### Participants

The present study was conducted in two medical and educational hospitals affiliated with the Iran University of Medical Sciences, Tehran, from January 2021 to February 2022, which are among the main centers for admitting patients with COVID-19. The first hospital was a public hospital with 30 ICU beds and an isolation room, and the second hospital was a semi-private hospital with 52 beds for patients with COVID-19. Patients in the critical stages of COVID-19 and requiring respiratory care were hospitalized in these departments. This study's participants were the family caregivers of patients hospitalized with COVID-19 in the ICU. Information about family caregivers and family members is provided in Table 1.

**Table 1:** Characteristics of the family caregivers and their family member.

**Caregiver**	**Gender**	**Age, y**	**Marital status**	**Education status**	**Relationship**	**Job status**	**Interview method**
CG1	Male	32	Single	Ph.D. candidate	Brother	Clerk	Face-to-face
CG2	Female	24	Single	Undergrad	Daughter	Student	Face-to-face
CG3	Male	32	Single	Postgrad	Son	Student	Virtual
CG4	Male	27	Single	Undergrad	Son	Unemployed	Face-to-face
CG5	Male	30	Married	Postgrad	Spouse	Assistance nurse	Face-to-face
CG6	Male	47	Married	Diploma	Son	Free job	Virtual
CG7	Male	45	Married	Undergrad	Spouse	Nurse	Face-to-face
CG8	Female	38	Single	Postgrad	Daughter	Doctor	Virtual
CG9	Male	28	Married	Postgrad	Son	Nurse	Face-to-face
CG10	Male	42	Married	Diploma	Spouse	Worker	Virtual
CG11	Female	33	Married	Diploma	Wife	House keeper	Face-to-face
CG12	Female	48	Married	Diploma	Mother	House keeper	Face-to-face
**Family member**	**Age, y**	**Gender**	**Education status**	**ICU lengs stay, day**	**Job status**		
FM1	52	Male	Diploma	5	Unemployed		
FM2	46	Female	Undergrad	8	House keeper		
FM3	54	Male	Diploma	3	Clerk		
FM4	60	Female	Diploma	10	House keeper		
FM5	25	Female	Diploma	12	House keeper		
FM6	62	Female	Diploma	15	House keeper		
FM7	42	Female	Diploma	7	House keeper		
FM8	58	Female	Diploma	10	House keeper		
FM9	50	Male	Undergrad	6	Teacher		
FM10	39	Female	Undergrad	20	House keeper		
FM11	33	Male	Undergrad	16	Engineer		
FM12	50	Male	Diploma	8	Military		

The participants of the study were selected based on their knowledge relevant to the research topic through a purposive sampling method (41). Given the researcher's previous familiarity with the ICU, a judgmental sampling strategy was employed to select participants who possessed a high level of knowledge and a deep understanding of the phenomenon under investigation. This approach was deemed necessary to achieve the goal of obtaining a deep understanding of the topic.

The inclusion criteria were as follows: the ability to speak Persian; being at least 18 years old; being at least a first-degree family member responsible for the care and follow-up treatment of the patient during hospitalization in the ICU; and being willing to talk about his/her experiences and perceptions. None of the included family caregivers were excluded from the study. All categories were discovered through sampling.

### Data collection

Participants were contacted by phone, and if they were eligible and desired to participate in the study, all details about the research were provided to them. Face-to-face or online interviews were conducted (using WhatsApp or Skype) based on the participant's preference.

Demographic information about family caregivers was collected with a questionnaire. In total, eight face-to-face interviews, three voice WhatsApp interviews, and one Skype semi-structured interview were conducted from January 2021 to February 2022 by researchers Rezaei, Shahrestanaki, and Milani. The interviews were conducted in a quiet place based on the preferences of family caregivers. Most of the family caregivers chose the meeting room in an ICU. Face-to-face interviews were conducted between the participant and the researcher, and non-participants were not present in any of the interviews. Before commencing each interview, the family caregivers discussed the purpose of the study and their desire to participate in it. After providing their consent, the time and place of the interview were determined. Additional interviews were conducted for two participants (numbers 7 and 11) to increase accuracy and clarify some ambiguity in creating questions or completing categories. Each interview started with a general question: “Tell us a bit about your experiences with your patient's infection with COVID-19.” Based on the first interview and the opinions of the qualitative researcher (TN), an interview guide was prepared and used for subsequent sessions (Table 2). Based on the answers of family caregivers, more probing questions were used, such as “Explain a little more?” and “Please give an example.” Interviews were managed and directed to collect information related to the study's main purpose. Interviews lasted ~39–68 min. Moreover, during the interviews, comprehensive notes were taken whenever possible from the transcripts during and after each interview to capture the participants' responses as accurately as possible. This allowed for the correctness and accuracy of the data to be verified by restating and summarizing the highlights of the participants' answers at the end of each interview. All interviews were recorded with the permission of the participants. The software MAXQDA version 10 was used for data analysis. In addition to the interviews, in this study, other data collection methods, such as field notes, were used as complementary and confirming methods for the interviews. The researcher's goal was to use the complementary method to collect data and fill the gaps in the category resulting from the data; therefore, to prevent misinterpretation of the data and capture their full depth, the researcher immediately began documenting notes in the field.

**Table 2:** Interview guide.

1. Tell us a bit about your experiences about your patient infection with COVID-19?
2. How do you describe this incident?
3. Tell me a little about the main challenges and problems of your patient?
4. Based on your experience, what challenges and problems were there during hospitalization and ICU period of your patient?

### Data analysis

Data analysis was performed simultaneously with data collection through the conventional content analysis approach described by Granheim and Lundman (42). The five steps of this approach include transcribing each interview verbatim, reading the text to understand its main ideas, determining and coding meaning units, classifying primary codes, and identifying the hidden content (42). The interviews were immediately recorded and transcribed by the researchers Milani and Shahrestanaki. The main researcher (the responsible author) read the transcripts of the interviews several times to gain a deep understanding of the data. In the open coding, words, sentences, and paragraphs related to the study's purpose in each interview were considered meaning units. Meaning units were coded using participants' words or suitable tags extracted from the data. The codes created by the researchers Rezaei and Ghezeljeh were discussed and compared continuously. To reduce items, similar items were merged and placed in a category. Among the 10 participants, no new materials or expressions were identified in comparison to previous participants, and all the concepts seemed well-defined and well-elaborated (41, 43). Then, two additional interviews were conducted beyond saturation, and the data of these people were placed in classes.

### Trustworthiness

The trustworthiness of the data was checked based on the steps suggested by Lincoln and Guba (44). In the present study, the participants were selected from family caregivers with maximum variation in terms of demographic variables to achieve credibility. Long-term engagement with the subject and allocating enough time to collect and analyze data made the data comprehensive and more accurate. The process of analyzing the data was prolonged, which involved transcribing the interviews, organizing and archiving the data, repeatedly listening to and reviewing the collected information, and coding and categorizing the data. Any disagreements by the research team were resolved by discussions until a consensus was reached. Moreover, data analysis was an iterative and back-and-forth activity as researchers moved from data collection to analysis and vice versa. Another way to ensure the validity of the findings was to review the data and findings from the analysis with the participants (member checking). For this purpose, in addition to returning speech and thoughts during some interviews, the researcher provided the complete typed text of the resulting codes to some participants. If they had valid suggestions, they were considered.

Moreover, during the review by the supervisor, the transcripts of some interviews, along with the extracted codes and emerging concepts and categories, were examined by the supervisor of the research project (2) to confirm and reach a consensus. The research team reviewed the interviews and coding methods to determine the reliability of the data. The implementation steps of the work were presented in detail in the methodology section of the research. The initial codes derived from the interpretation of the participants' experiences and examples of how to extract categories and excerpts from the text of the interviews were presented for each of the categories so that the external observer (Peer Check) could perform the audit according to these documents and achieve the objective of the study. In the present study, for confirmability purposes, data were extracted from the participants' conversations, and the researchers set aside their views and motivations. The confirmability of the study was ensured by researchers by using the documents used in all stages of the research. Moreover, obtaining the opinions of experts in this field was one of the other factors that ensured the confirmability of the data. For this purpose, the data, codes, and secondary categories were provided to professors and experts in qualitative research. Based on their corrective opinions, the necessary changes were made, external control and review were performed on the data, and the verifiability of the data was made possible. To ensure the transferability of the study, the researchers explained all the details of the study, including the demographic characteristics and study context, the selection of participants, the collection of information, and the process of data analysis in a clear and specific manner. The findings were presented in a comprehensive and rich manner, and appropriate quotations were used. Additionally, the steps of conducting the study and the activities carried out in the course of the research were precisely written with a clear and purposeful description so that others in different places could do it.

### Ethical considerations

This study was approved by the Ethics Committee of the Iran University of Medical Sciences, Tehran (IR.IUMS.REC.1399.1034). While explaining the study's objectives to the participants, they were assured that their information was confidential and that confidentiality was ensured at all stages of the study until the report was submitted. In addition, informed consent was obtained from all participants before each interview. All the methods were conducted according to the relevant regulations and instructions. We took the necessary measures to safeguard the intellectual property of this study and can confirm that these steps were implemented. We followed our institution's regulations regarding intellectual property. We also confirmed that any aspect of the study covered by this manuscript involving experiments on humans was carried out with the ethical approval of all relevant authorities and that such approvals were acknowledged in the manuscript. It was also confirmed that all procedures were carried out in accordance with the relevant guidelines and regulations. At each stage of the research, participants had the right to withdraw from the study and were assured that their withdrawal would not disrupt their loved ones' treatment. All the text of the interviews and the information of the participants were stored on a flash drive with a responsible author to ensure confidentiality.

## Results

Twenty-eight family caregivers invited to the interview were deemed eligible for the study. Sixteen subjects did not participate in the study, citing reasons such as work and ongoing involvement in patient care. Therefore, interviews were conducted with 12 participants. Ten of them had a single interview, and two others were interviewed three times. The average age was 35.57 years. Approximately 75% of the participants were men (three husbands, four sons, and one brother) and 25% were women (one daughter, one mother, and one wife) (Table 1). The participants described their experiences with the hospitalization of their family members in the ICU. Table 3 shows the categories included in this study: the hardship of care trajectories, pre-loss mourning, and contributing factors in resolving family health crises.

**Table 3:** Categories, sub-categories, and some quotes of the participants.

**Category**	**Sub-category**	**Participants' statements**
The hardship of care trajectories	Immersion in unknowns	“*When a patient is hospitalized in ICU, the patient's condition should to be updated moment by moment… we need have a connection with the ICU to know what is happening to the patient. It will reduce psychological pressure for family caregivers. When I called on the phone… the nurse always said he is fine, when she talked me only 2 minutes! That's it!… I was expecting them to explain for me… He is in ICU, and it is not a normal condition that I can call my patient with my mobile phone… Even little news about the treatment process could be a lot of news. It will be a pleasure for us… I think this is a missing link in the ICU department, that when the patient enter ICU, all data is unavailable for the patient's family…” (P3) “Sometimes I say to myself that I wish I hadn't transferred my father from our city… It would be better if I hospitalized him in our city. It was bad for him to move… I was in Tehran and I couldn't leave my job… I said that I would bring him to Tehran so that I could be with him… It was very difficult for me to make a decision, but in any case, Thank God, a bed was found, and we have admitted Dad…” (P11)*
	Lack of caring facilities	“*The hospital really didn't have any facilities… The hospital laboratory informed us that our device was broken, and they couldn't measure oxygen content in the blood… For the past 2 weeks, they were calling me from the hospital; I went by car and they gave me a vial to take it to the laboratory out of the hospital… do the test there… bring us the answer… that is what I did twice a day…. It was really awful, and I think they had facilities only for CBC measurement! Their medicine is nothing! I could never get medicines from my hospital pharmacy. I always went to pharmacies outside…” (P3) “They said that your patient should get CRRT. We got two filters from a store that they introduced us. Filters were very expensive, but we think that he is young, it's a shame to die young. We will borrow from a factory where my brother was employed…” (P12)*
	Negligence in caring	“*They talked me so way that I was always concern that they might call me that my patient has died. Immediately after entrance of the patient in ICU, these things happen, I asked myself whether they really care the patient or not; they considered patient as a dead. It may not be matter to anyone if he gets better or not.” (P9)*
	Ignoring families by health care providers	“*Now, for example, my mother's doctor is walking in front of me, I don't dare to ask him at all…” “Because he says that you are prying into my work…” “Well, I say that if I don't interfere, you won't notice…” “I came from half past five in the morning and stood behind the ICU until seven o'clock when the doctor came to visit my mother…” (P8)*
	Self-ignorance	“*There was so much pressure that after a while I felt that I no longer had my knees and I could not walk…” “I was exhausted physically. It's like a tsunami is following you, but you can't walk anymore…” “But I'm not upset at all, I just want to deal with all these hardships to see that my patient is getting better…” (P7)*
	Perceived stigma	“*Another challenge is that other families think that the issue of Corona is similar to leprosy, which means that they will cut off communication with you… Of course, it may be logical, but it makes an unpleasant feeling for you. It makes people wonder, the neighbors think that all family members have corona.” (P1)*
Pre-loss mourning	Emotional and psychological turmoil	“*My mother sometimes says that when you came home, I knew what you were like… it was obvious from your confused face that your father was not well… maybe 80% of this confusion was because I didn't know about the condition of my patient, and what is going to happen to my patient next week? It was one of the worst possible situations for me….” (P3)*
	Witness the exhaustion of loved ones	“*I saw that my wife was confused and took out the NIV, but they didn't give it to her. Her saturation dropped to 60. She was very restless and keeps getting off the bed. When I got to see her, her head was cyanotic, like a dead…” (P7)*
	Separation suffering	“*We are waiting for this door to be opened and either his body come out or he come out safe… this waiting bothered me a lot and will remain on my mind for the rest of my life.” (P3)*
	Fearing of loss	“*When my wife was admitted to the ICU, dialysis was applied for patients next to her, and often death happened next to my patient, this death and dialysis and… disturbed me and my wife in terms of psychological balance. I was always worrying that my patient might end up like this…?!” (P10)*
	Anticipatory grief	“*The fact that he come out of the ICU to talk us or to walk with him again, is a miracle. Rather than coming out more easily, we expect that, for example, they will give us bad news; your patient has worsened, or he has gone under the machine, or things like this…” (P9)*
	Blame the disease causative agents	“*The vaccine came too late. First, they said that we produce it ourselves, there is no need to import any vaccine. When the disease spread everywhere, and they lost control… Other countries gave the third dose of vaccine to all age groups, but our children and elderlies didn't still get the first dose… it's really a pity…” (P6)*
	Perceived helplessness and despair	“*The family members didn't come forward until they found out that my brother's condition is serious, and they didn't get involved, especially his wife's family. I am a woman. There is no one to help me. At least someone came and get his medicine or prepare a meal for him. I am fearing of getting sick, I do not drink water or eat food in the hospital…” (P12)*
Contributing factors in resolving family health crises	Individual role of family caregiver in health engagement	“*As soon as I realized that my lady is not well, I quickly followed up her treatment and hospitalization… I called all my friends who were in the treatment team, and finally a special bed was quickly found for her, and we admitted her… I started looking for medicine. I asked all my friends and family to look for medicine… I even asked my friends abroad to send me medicine…” (P11)*
	Role of professionals in health engagement	“*The treatment staffs present in a center where there are corona patients, high amount of virus is there, and they are working. I really understood how hard they are working. Sometimes their good behavior caused calmness…” (P1)*
	Role of interpersonal factors in health engagement	“*My wife's friend helped us a lot, she helped us even more than my family… she prepared all kinds of food and sent us to the hospital… I was worried about my son who is at home, but my wife's friend didn't let my son feel any shortage while his mother was in the hospital…” (P7)*

### The hardship of care trajectories

The first category describes the kind of problems that family caregivers face during a family member's illness. They had never experienced such problems in their lives, had limited time to address them, and were alone in the process. In addition, caregivers had to face unknown events and needed support. There was no supportive environment, and they may have had to make continuous efforts to solve those problems.

#### Immersion in the unknown

The family caregivers faced numerous unknown events, including a lack of knowledge about the patients' prognosis and treatment processes, limited access to doctors for updates on the patients' health status, insufficient information about medications and care equipment, and inadequate guidance on managing the patient after discharge. The use of home care equipment was something about which family members knew little. Furthermore, families had difficulty making decisions. Is it the best decision to admit the family member to a nearby hospital, or is it better to transfer to another center? Is the sale of a house or a car a priority over the purchase of a foreign drug, or are Iranian drugs sufficient? Is it permissible to transfer the patient from a small city to the capital? These were all questions that family members were faced with and were confused about when making the right decision.

P1: “We don't know how long he will be hospitalized; we don't know the complications. This is stressful. We don't know the best trainings for corona. Now…, we are thinking that if today he should be discharged, what precautions should we take after that? Now, we all say we don't know, what about the later? What the nurse told us was that I have to provide devices such as oxygen generators, I don't know how to rent them; I asked them that their prices are very high and how to take care of them; these are all the tensions that I really think. They can be reduced with a more correct education for the family members. The most important thing is being far away to our dear and not seeing him anymore. It is very hard, and we don't know what his condition is. We don't know how his condition is!!?”

P9: “When they started taking some drugs like Actemra for my father, they said that get foreign drugs, or we will use a native type. Regarding the injection of Iranian medicine, they also said that you have to agree that we want to inject this medicine, it has complications; the patient may get bradycardia, I don't know that which complications can happen to him, he has cardiac arrhythmia. But the cost of foreign medicine was expensive, it was a bit difficult. His stress that we agreed to inject Iranian medicine was a very bad experience.”

#### Lack of care facilities

Participants expressed that they attempted to achieve minimum care facilities during the hospitalization. Finding a bed in the ICU, providing medicines, and even a lack of diagnostic facilities in a medical center put significant levels of pressure on the family members and caused mental and physical distress. Furthermore, family involvement in the treatment process resulted in significant financial burdens. The high costs associated with medicines, medical equipment, and hospital beds, combined with inadequate insurance support, forced many families to resort to borrowing from formal and informal sources. These difficulties were all endured with the hope of a recovery for the family member, but there was mostly uncertainty about the outcome.

P2: “We didn't have a problem until they told us 2 days ago that you have to get an IVIG. We were very upset… how is it possible for a hospital to tell the patient to go out of the hospital to get IVIG? You would find it outside, but it's very expensive, go find it and get it for your patient. The drugs prices were increased 10 times than beginning of Corona… It is clear that there is a mafia behind it…. My friend has MS and before Corona beginning, he bought IVIG for 100 thousand Tomans. It's now 10 times more expensive…!!!”

P1: “Now we found an empty bed in a private hospital. Each connection certainly take money from us, ambulance, supervisor, and… At the beginning of admission, they charged us a heavy fee, and for now, the fees are still heavy, we are paying the fees.”

#### Negligence in care

Family caregivers believed that medical staff failed to properly care for their family members. Low levels of responsibility, the occurrence of numerous errors throughout the treatment process, a lack of confidence in meeting all physical and mental needs of the patient, insufficient knowledge and skills of healthcare personnel, low importance of patients' fate among healthcare personnel, and the hopelessness of patients' recovery were among the issues reported by the participants.

P3: “…I insisted a lot until I finally informed that my father is very sick and needs to be in ICU…maybe if I didn't insist and follow up…that doctor had an irresponsible attitude…he just said yes! Your father CT is bad! He should be hospitalized…but no change was made…”

P7: “… What happened, and I found out after a lot of research that when my wife was admitted to ICU, she was very sick at the first night, she could not catch her breath because of her pregnancy… she was suffering from shortness of breath. The nurse was sleeping, and her sleeping caused this to happen, and the drugs were injected more or less. You see, in this disease process, there are many items that cause the patient to get sick or get better or die. One of the most important factors is human resources, including work commitment, work rules, responsibility, knowledge, and experience. I think that their staff, at least in the night shift, had low commitment… maybe because they are younger or less control is applied in the night shift…”

#### Neglect of families by healthcare providers

Family caregivers stated that they were willing to participate in the treatment and care decision-making process for their family members. They attempted to establish a constructive relationship with the medical staff. They also attempted to win the staff's trust in different ways to obtain more information about their loved one's condition. However, this communication was not possible for various reasons.

P3: “The caregiver goes to meet the patient's doctor and asks him what about the condition of my patient?” In such cases, a person wants two things… Either they tell that his condition is getting better or that his condition is getting worse… I have always heard from doctors that We don't know!… Is he okay?… We don't know!… How will it be tomorrow?… We don't know!… When I returned home, my mother asked me: how about your father? And I kept saying, I don't know… I didn't know what to answer…”

#### Self-ignorance

The family caregivers reported facing numerous difficulties and challenges when attempting to meet the needs of their loved ones, often neglecting their own needs in the process. Carrying the burden of care alone, enduring adversities and unkindness, becoming involved in their loves ones' care, experiencing fatigue and insomnia, and having to borrow and ask for financial help from others were among the problems that family caregivers had to deal with.

P8: “We came here in the morning…”. “For 36 days, our lives have been completely abandoned…”. “We don't think about ourselves at all…”. “I am looking for medicine every day and sleeping in the car at night…”.

#### Perceived stigma

The family caregivers expressed concerns about being ostracized or blamed by others, which added to their psychological burden. This situation led them to feel isolated and attempt to keep the problem hidden. They attempted to solve the problem themselves.

P7: “Corona has changed a lot of things. Many good things became bad, and many bad things became good. A person who was close to you did not know you now. He doesn't even come back to the hospital to check your patient's condition.”

### Pre-loss mourning

The second category describes the emotional problems of family caregivers and family members. Family caregivers exhibited behavioral and emotional responses to the possibility of losing a loved one, and they mourned in private. Requesting a meeting with the patient to read the Adilleh prayer (a special prayer that Muslims recite at the bedside of their loved ones to make it easier for them to die and not suffer pain), contacting relatives to seek forgiveness for their loved one, inquiring about the price of the grave, and holding a funeral ceremony for a family member were among the things that showed that families were preparing themselves for the possibility of losing a family member.

#### Emotional and psychological turmoil

Family caregivers reported experiencing high levels of anxiety, stress, and fear from the moment of their loved one's admission to the ICU, feeling mentally confused and uncertain about the path of recovery for their family member, questioning whether the treatment plan was appropriate and if all necessary resources were available. The unknown future of their loved one's health was a constant source of worry for the participants, leading to feelings of uncertainty and fear about what to expect. “I'm a doctor myself… You can see that there is a lot of lung involvement. OK; He comes back sick. At first, you will be very anxious, but in the end, you may reach despair.”

#### Witnessing the exhaustion of loved ones

The experience of watching a family member endure physical and mental hardships caused distress for the caregivers in this study. They were troubled by the lack of measures taken to alleviate their loved one's discomfort. The sight of multiple wounds on the patient's face caused by the use of non-invasive respiratory ventilation (NIV) masks, the distress of the shortness of breath, the absence of peace and comfort for the patient, and the cries and screams of the patient due to restraints and confinement evoked feelings of powerlessness among the caregivers.

P2: “It was clear that mom needed ICU. Every hospital has a level of care, and I knew that she needed a higher level of care. Mom even couldn't move. When [sic] she moved, a severe oxygen drop occurred. It was clear that he needed ICU, as she didn't have the strength to breathe…”

#### Separation suffering

Participants stated that their separation from their family members and his/her feelings of loneliness and abandonment in the ICU made them anxious. They were worried that they might not be able to see their loved one again and that they would not be able to help their patient when he/she needed them. Even when they were allowed to meet their family member in the ICU, they were worried that this might be the last meeting.

P1: “Another problem is that when the patient is in the ICU, visits are prohibited; when a patient comes to the ICU, he must see another person; there is a mental pressure that you don't know about his mental state and his condition, and on also he is worried about our condition.”

P4: “The hospital staffs [sic] did not allow you to go to talk, see, or touch him to reduce his stress. He needs to think that there is someone who is paying attention to me…”

#### Fear of loss of loves one

Witnessing unpleasant events such as the deterioration of other hospitalized patients, the deaths of patients, carrying off their bodies from the ICU, and observing the mourning of their families in the hospital or experiencing the loss of relatives due to illness scared the family caregivers. The fear of hearing bad news at any moment caused them anxiety and robbed them of the motivation to continue.

P6: “Fear came to me so many, I said that things that happened to many others, which were unfortunate, might happen to me too. I saw that many people had lost their family member here, many of them were young. Everyone was trying to return, but it was not possible. It was very bad that a patient came to hospital on awake, but his family saw that the patient collapsed.”

#### Anticipatory grief

The family caregivers mentally prepared themselves for the worst-case scenario, and they found themselves contemplating life without their loved one's presence. Seeing his or her loved one's pain, the family caregivers wished for his or her loved one to be free of this situation as soon as possible, even if it implied death.

P12: “I know that my brother will not live (crying), and we have to think about the cost of his shroud and burial, but I am concerning about his little children. How do they grow up without their father? They don't have any supporters… God bless them…”

#### Blame related to the disease causative agents

The family caregivers listed factors contributing to the crisis in the family and often engaged in bargaining and questioning why this problem occurred for their family?!… How did he get infected?!… In addition, family caregivers may go over some possible reasons in their mind. If there was a vaccine, this would not have happened!! This would not have happened if people had complied!

P9: “People don't believe in the disease… They still have parties and trips… We are thoughtless people; all misfortunes are because of ourselves… My father had not seen his brother for 2 years because of Corona. Now he got Corona because of people's thoughtlessness…”

#### Perceived helplessness and despair

The family caregivers experienced conflicting emotions during the hospitalization of their sick loved ones. When he/she heard the good news about the clinical condition of his/her family member, he/she was happy and conveyed this happy news to all family members. When he/she heard bad news about the patient's condition, he/she did not answer any phone calls and preferred to do everything himself/herself. Finally, the caregiver felt that he/she was alone and felt helpless.

P7: “I have never been in the position of a caregiver… We sisters are really crippled. We are desperate. There is no one to help us… I don't have a father, I don't have a brother, our family is far away from us, and we are really getting annoyed…”

## Contributing factors in resolving family health crises

The third category shows that in addition to the problems and concerns encountered by the family caregivers, several factors were identified as being particularly instrumental in resolving the health crisis of the affected family member. These factors include the following aspects: (1) The primary family caregiver who assumed the major responsibility for caring for the affected family member; (2) healthcare workers who provided care for individuals with the disease and helped families navigate the crisis, despite the dangers posed by the disease and limited access to personal protective equipment; and (3) the active participation and support of the affected family members' relatives in addressing the crisis through mutual assistance and collaboration.

### Individual role of family caregivers in health engagement

The family caregivers cared for their ill family members and attempted to be involved in all stages of treatment. He/she addressed the physical and mental problems that the family members were dealing with and provided emotional support and encouragement to the patient and other family members. He/she also provided all types of facilities. He/she attempted to be strong and firm in the face of problems and assured the family member that all facilities were ready outside the ICU. Therefore, the family caregiver plays a vital role in managing the family's critical situation.

P3: “In addition to the fact that your loved one is in the hospital bed with a lot of troubles, you are responsible for giving peace to the family, and I didn't want to transfer this mental pressure to the family. I was trying to improve the environment with gentleness and hope.”

### Role of professionals in health engagement

The participants mentioned that healthcare workers played an important role in helping families navigate the health crisis. Caregivers stated that they trusted the healthcare providers throughout their loved one's hospitalization. Providing support and instilling hope in family members and fostering positive interactions with them instilled confidence and trust in the treatment staff. Family members felt that their voices were heard and that they were not alone. Support from healthcare providers gave the family caregivers hope and encouragement.

P10: “In my opinion, nursing, medicine, and equipment are three most important factors in achieving the desired result in the recovery of my patient…”. “I believe that the role of human resources was very important in the death or recovery of patients…”. “If treatment staff had not worked for my patient, I couldn't find any motivation to get my family out of the crisis…”. “I really didn't know what to do, who to ask for help…”. “It was the treatment staff who showed us the way and the well way…”. “When I think to myself, I can't put myself in the place of nurses for a moment, because they really care for patients with all their heart…”.

### Role of interpersonal factors in health engagement

The participants reported receiving support from people around them in the form of various resources. These included financial aid and assistance in caring for other family members. Moreover, participants noted that families facing similar circumstances provided mutual support and understanding, for example, by sharing information about obtaining rare equipment or medicine and providing each other with practical assistance in achieving their goals.

P2: “We are Turkish; we have a lot of family… We have a lot of affection [laughter]. Now they are calling me and bothering me… they all ask about my mother condition… We are not at home right now; we are at house of our relatives. Even though I might be a disease carrier, my father might be a disease carrier… We said that we should stay in a hotel, but our relatives didn't let us.”

## Discussion

This study's findings revealed that family caregivers experienced unprecedented challenges during the hospitalization of their family members with COVID-19 in the ICU. The personal narratives and stories told by family caregivers may shed light on the truth that has so far been overlooked.

These families made significant sacrifices, both physically and mentally, to save the lives of their loved ones and went to great lengths to provide comfort to their families. The present study aimed to examine the experiences of family caregivers during the illness and hospitalization of a family member in the ICU. The findings were organized into three main categories, which include: (1) the hardship of care trajectories; (2) pre-loss mourning; and (3) contributing factors in resolving the family's health crises.

The hardship of care trajectories is one of the categories that was extracted from the data, indicating the occurrence of a psychological crisis in the family. Family caregivers experience problems when a family member is sick and hospitalized, and facing these problems consumes time, energy, and all of the available support resources. However, in this study, due to unpredictability and the deadly nature of this disease, sufficient time was not available to resolve these problems, and the inability to solve these problems inevitably led to tension within the family.

Immersion in the unknown is a subcategory of the hardship of care trajectories. This subcategory highlights that, after the ICU doors were closed, all family caregivers' means of communication with their loved ones were severed, leaving them alone with numerous questions and concerns about the future. They did not know what fate awaited their family members. In line with this finding from the present study, it was found in Lissoni et al.'s study that being unaware of the condition of the sick family member hospitalized in the ICU can evoke a sense of fear and anxiety among family members (45). One of the reasons for the occurrence of psychological reactions such as fear and anxiety in family caregivers is the lack of clinical information on family members, and it seemed necessary to inform and educate families when a family member was hospitalized and struggling with a life-threatening disease. Therefore, healthcare workers should provide the family members of the patient with the required information.

One of the most important concerns that family caregivers faced during this outbreak, according to the Immersion in the Unknown subcategory, was the need to make decisions about the unknown. Indecision and confusion in determining the best treatment led to feelings of despair among the family caregivers. As the selection of different treatments for the patient was often left to the family, and the treatment staff was uncertain about the treatments, the family members were required to sign a consent form for the treatment of their loved one, and the assigned physician was uncertain about the course of treatment. These were times of uncertainty, and they created doubts about the quality of care among the family members. This added to their uncertainty about the quality of care for their loved ones. With other life-threatening illnesses, treatment staff and families typically have more reliable options and more time to make decisions about the type of treatment. However, it was not the same case for COVID-19. During this outbreak, access to treatments was highly limited, and this problem and the uncertainty caused psychological pressure for the family members. However, in the case of an acute and lethal disease such as COVID-19, the need to decide on or access treatments was highly limited. This decision to choose an uncertain treatment was entrusted to the patient's family, which resulted in significant psychological strain on the family. Noome et al. found in their study that with other diseases in which patients needed special care, family caregivers were afraid of choosing a treatment plan for their family members. The participants also reported a preference for leaving decisions to physicians, citing feelings of isolation and a lack of support during the decision-making process (46). This sense of loneliness in the decision-making process in the family of hospitalized patients with COVID-19 was much higher because they were the final decision-makers regarding the type of treatment, and the treatment staff did not have enough confidence in the effectiveness of the treatment plan and started the treatment plan for the patient based on the family's choice. Thus, feelings of isolation and uncertainty about choosing the appropriate course of treatment for a sick family member were some of the most significant challenges faced by the family members. This sense of uncertainty about making decisions was more commonly reported, especially in the pandemic's early stages when there was little information about the nature of the disease.

During the COVID-19 outbreak, nearly all medical centers were faced with shortages of essential care equipment such as beds, medications, and even diagnostic equipment. The acquisition of basic equipment was often left to the patient's families. Families were asked to buy rare medicines at exorbitant prices. They had to exert great effort to secure even the most basic diagnostic tests and care facilities for their loved ones. Other studies showed that providing these necessities was a significant challenge for family caregivers. They spent most of their time at pharmacies and medical equipment centers instead of caring for their family members (30, 46). According to this finding from the present study, care and treatment equipment were assigned to family members. This study also found that family caregivers experienced a significant lack of confusion due to the challenging health conditions of their family members and the uncertainty surrounding the authenticity of drugs and equipment procured from untrustworthy sources, which resulted in a range of emotional and psychological responses among the caregivers.

Another challenge faced by families during the COVID-19 pandemic was the financial burden caused by the disease, including the cost of purchasing expensive medications. They were under severe financial pressure to save their loved ones' lives. This finding is consistent with that of a study by Mottagi et al. which found that, during the pandemic, families faced financial strain due to the loss of income and increased costs of treatment for their loved ones (38). This represented an additional economic burden on families, which is considered one aspect of the burden of care. In other diseases, such as cancer, it has been observed that this aspect of the burden of care can negatively impact the quality of life of family caregivers (47). Another challenge faced by the families was the lack of attention from healthcare providers. The family was often excluded from the decision-making process and was provided with little information about their loved one's condition, which led to conflicts between the family and healthcare providers. In a study by Picardi et al. it was found that one of the unmet needs of families was communication between the family and treatment staff due to incomplete information about the health status of their family members (41). The clinical experiences of researchers and the care of patients with COVID-19 showed that receiving incomplete information about the family member's health conditions and the lack of effective communication between the treatment staff and the family members caused them to become angry and hostile toward the treatment staff.

The participants also reported instances of negligence and malpractice by healthcare workers while caring for their sick family members. The participants cited low commitment and accountability of care workers and inadequate knowledge and skills of staff in managing the disease as areas of concern. The study by Chen et al. discovered that family members considered the healthcare system accountable for the deterioration of their family member's condition due to delays in starting treatment. Moreover, family stress levels were exacerbated when healthcare providers failed to diagnose COVID-19 in their loved ones despite the presence of alarming clinical symptoms (48). One possible explanation for this finding is that family caregivers often observed nurses providing care to their loved ones with limited experience in managing the symptoms of the disease. Many hospitals had to hire inexperienced nurses or nursing students due to a shortage of staff, and many nurses quit their jobs during the outbreak. This shortage of skilled nursing staff may have led to a negative perception among families of patients with COVID-19 hospitalized in the ICU, leading them to believe that the deterioration of their loved one's health was due to the carelessness of the healthcare personnel.

Family caregivers experienced a range of hardships and challenges. They prioritized the needs of their family members over their own, resulting in unmet needs such as lack of sleep and rest, poor dietary habits, anorexia, and digestive issues. Earlier studies indicated these families' physical and mental problems, such as insomnia, anorexia, worry, and despair (20).

The study found that the perceived stigma caused by hiding the family member's illness out of fear of rejection from others was a problem in the treatment process. This stigma was described by Rahimi et al. in their study as an unpleasant social factor that family caregivers faced. They withdrew from the community and avoided social interactions out of fear of being labeled as carriers. This isolation led to problems such as psychosomatic disorders among the family members (49).

Pre-loss mourning, which emerged as the second category from participants' experiences, was related to emotional crises among the family members. Due to the high mortality rate of COVID-19 among patients in the ICU, family members were constantly expecting bad news about their loved one's condition and even anticipated their death. They displayed behaviors that indicated their readiness to mourn. Fears, worries, stress, and anxiety due to the unknown fate of the family member led to mental and emotional distress in other family members. In the study by Rahimi et al., family caregivers described such experiences as highly difficult and terrifying. Different experiences were reported, but the most common were fear, anxiety, worry, sadness, despair, uncertainty about the fate of their loved ones, and feelings of helplessness about the condition, all of which led to mental distress within the family (49). Experiences related to loss and bereavement in acute and fatal diseases such as COVID-19, where people do not have sufficient time to process the impending loss, likely have more complex consequences than the loss of a family member with a chronic illness such as cancer. With diseases such as cancer, family members have more time to accept the loss and adjust to it, and they are more emotionally and psychologically prepared. The consequences of losing a loved one to COVID-19 caused more problems than chronic, incurable diseases such as cancer.

The family caregivers saw the equipment and respiratory aid devices as well as the struggle of their loved ones while breathing. This gradually caused feelings of fatigue and weakness, making it difficult for them to witness their loved ones' pain and physical limitations. They wished to make the patient comfortable as soon as possible, even with death. In the study by Chen et al., participants described how their family members struggled with the complications of COVID-19, such as persistent shortness of breath, pain, and post-traumatic stress. The unpredictability of a family member's health caused anxiety for family members and made it more intense (48). The results of the present study and the study by Chen et al. revealed that, although patients suffer high levels of physical and emotional pain, the pain and suffering are more intense in conscious patients who have progressive breathing problems. Moreover, family caregivers are also as distressed as the affected family member. They suffer emotional pain and suffering, which makes the treatment process more complicated.

Separation suffering was yet another prevalent problem among the participants, with almost all reporting feelings of longing, abandonment, and loneliness following separation from their family members in the ICU. This sense of disconnection and failure to reunite with their loved one led to significant levels of anxiety and feelings of separation among the family members. Previous research established that the loneliness experienced by having a family member in the ICU might evoke feelings of helplessness and guilt among other family members (20, 30).

The fear of loss was commonly reported among the majority of participants. Witnessing the deaths of other patients with the same conditions, the high mortality rate of patients with COVID-19 reported in the media and even the deaths of close relatives led to a sense of disappointment within the family. These findings were in line with the study by Bartoli et al., in which families experienced the fear of loss after seeing their family members hospitalized in the ICU. Seeing family members connected to different devices with a low level of consciousness or receiving high doses of drugs made this feeling more intense (20).

The family caregivers were found to be attempting to mentally prepare themselves for potential deterioration in the health conditions of their family members or even for the possibility of their absence in the future. This finding of anticipatory grief is consistent with the study by Rahimi et al., who stated that family members of dying patients initially considered the diagnosis of COVID-19 equivalent to death, and some families even experienced anticipatory grief after the recovery of their family member (49).

Family caregivers expressed sadness by blaming the causes of their family member's illness. Resentment toward the ignorance of people in the community for not following the protocols, frustration with delays in accessing the vaccine, and anger toward distressing news in the media were some of the emotions expressed. This finding of the present study confirms the findings of previous studies that revealed that family members responded to a loved one's diagnosis of the disease by blaming themselves for its spread to other family members, blaming medical staff for the late diagnosis, or blaming themselves for not following preventive protocols (30, 36, 48).

The family caregivers reached a state of helplessness and desperation during the illness and hospitalization of their loved one in the ICU. They experienced loneliness and helplessness, and their relatives withheld help from the family of the patient with COVID-19. In previous studies, it was found that friends and acquaintances rejected family members due to the presence of a disease in one of the family members, and people around them refused to contact them because the family members were perceived as disease carriers. This made the family socially isolated and lonely (20).

Despite all the hardships faced by families who had a loved one battling COVID-19 in the ICU, some factors helped the families manage this health crisis.

Under the category Contributing Factors in Resolving the Health Crisis, family caregivers identified their desire to save their family member's life as a driving force. Therefore, families made significant efforts to address the health crises of their loved ones.

The first contributing factor in solving the health crisis was the key role of family caregivers in resolving the health crisis. The family caregivers were responsible for monitoring the treatment and care of their loved ones. In addition to caring for the hospitalized family member, the family caregiver also had to care for other family members who had a milder illness, maintain the psychological wellbeing of the family, and follow up on the treatment of the affected family member. The second contributing factor in solving the health crisis was the role of professionals, including doctors and nurses, who were on the front lines of the fight against COVID-19 and spared no effort to provide comfort and convenience to the families. The healthcare professionals attempted to contribute to managing the family member's health problem by having empathetic communication with the families and honoring the family's voice as an advocate. They defended the physical and mental health of the families to the best of their ability. In the study by Chen et al., it was reported that family caregivers appreciated the efforts and sacrifices of the healthcare personnel for their family members and stated that healthcare workers treated them and their family members respectfully (48). The third contributing factor that helped mitigate the health crisis was the role of interpersonal factors, such as the beneficial support received by families from their neighbors and relatives, including financial and emotional support. In addition to family members, other families with hospitalized patients played an important role by providing mutual assistance and sharing their experiences with families in similar situations.

### Strengths/limitations

This is a multi-centered study that included participants with different health conditions. The researchers aimed to include participants from both public and private hospitals with varying economic statuses and levels of access to health services. Another strength of this study was its focus on the experiences of family caregivers of patients with COVID-19 admitted to the ICU. This highlights the importance of considering the role of family caregivers in the recovery of their loved ones, even those battling other life-threatening diseases. One limitation of this study is its inability to conduct face-to-face interviews with all participants due to social distancing protocols. This made it difficult to observe the emotional states and reactions of participants in a virtual interview or to observe the context of their experiences with COVID-19. Another limitation of the study was the difficulty in accessing participants and coordinating interview times due to their heavy involvement in follow-up treatment and the preparation of medicine for their loved ones, which resulted in frequent cancellations and postponements of interviews.

## Conclusion

The findings of this study highlighted the experiences, perceptions, and feelings of family caregivers whose loved ones had a life-threatening illness. The COVID-19 pandemic brought forth an unprecedented challenge as it was an unknown disease. In such a scenario, the role of patients' families in the treatment process became crucial, more so than any other factor. The treatment process was unpredictable and uncertain, and families had to actively participate and take all the measures necessary to save the lives of their family members. However, families suffered a great deal of financial, physical, and psychological distress. They were forced to sell their assets to provide limited treatment facilities for their loved ones. They prioritized their physical needs over their own and experienced fear, worries, stress, anxiety, and a high level of uncertainty, which caused them to fear losing their family member. Despite receiving support from both their social networks and the healthcare system, families found that these resources were insufficient to meet their needs for effectively managing the health crisis.

The results of this study highlight the importance of considering the role of family caregivers in treatment plans, and healthcare personnel should be aware of the support of these families need to meet the need their patient's needs. Acknowledging and addressing the needs of families facing such serious disease can help them better cope with the disease. Building trust and fostering a strong relationship with the families of their patients can enhance their capacity to deal with pandemics or other diseases with limited treatment options.

Our findings from this hypothesis-generating study emphasize the need for further research on long-term support plans for family caregivers of critically ill patients in the ICU. We recommended that future studies include larger and more diverse sample sizes to validate this study's findings.

## Data availability statement

The original contributions presented in the study are included in the article/Supplementary material, further inquiries can be directed to the corresponding author.

## Ethics statement

The studies involving human participants were reviewed and approved by Iran University Ethics Committee (IR.IUMS.REC.1399.1034). The patients/participants provided their written informed consent to participate in this study. Written informed consent was obtained from the individual(s) for the publication of any potentially identifiable images or data included in this article.

## Author contributions

TN was as research team supervisor and helped in data saturation and revised final manuscript. All authors contributed to the article and approved the submitted version.
