# Supplementary material for: Exploring family caregiver challenges in caring for patients with COVID-19 in intensive care units
Source: Front Public Health. 2023 Mar 9;11:1057396. doi: 10.3389/fpubh.2023.1057396 (PMC10034162; doi:10.3389/fpubh.2023.1057396)
Supplement: Supplementary file 1 [file Data_Sheet_1.docx]

| **Them and Subthemes** | | **Interviews** | | | | | | | | | | | |
| --- | --- | --- | --- | --- | --- | --- | --- | --- | --- | --- | --- | --- | --- |
|  |  | 1 | 2 | 3 | 4 | 5 | 6 | 7 | 8 | 9 | 10 | 11 | 12 |
| **Pre-Loss Mourning** | Emotional and Psychological Turmoil | * |  | * | * | * | * | * | * | * |  |  | * |
|  | Witness the exhaustion of loved ones |  | * | * | * | * |  | * | * | * | * |  |  |
|  | Separation Suffering | * | * | * | * | * | * | * | * | * | * | * | * |
|  | Fearing of Loss | * | * | * |  | * |  | * | * | * | * | * | * |
|  | Anticipatory Grief | * |  | * | * |  |  |  | * | * |  | * | * |
|  | Blame the Disease Causative Agents |  |  | * | * | * | * |  |  | * | * | * | * |
|  | Perceived Helplessness and Despair | * | * |  |  |  |  | * | * | * |  |  | * |
|  | | | | | | | | | | | | | |
| **The Hardship of Care Trajectory** | Immersion in the unknowns | * |  | * | * |  | * |  | * |  |  | * | * |
|  | Decision with Uncertainty |  | * | * |  |  |  |  |  | * |  | * | * |
|  | Lack of Caring Facilities | * | * | * | * | * | * | * |  | * | * | * | * |
|  | Ignoring the Family by Health Care Providers | * |  | * | * |  |  | * | * | * | * | * | * |
|  | Self-Ignorance |  |  |  |  | * | * | * | * | * | * |  | * |
|  | Perceived Stigma | * |  |  |  |  |  | * |  | * |  |  | * |
|  | The Experience of Negligence in Care |  | * | * | * |  |  | * | * | * | * | * | * |
|  | Financial Repercussions | * | * | * | * | * |  | * | * | * | * |  | * |
|  | | | | | | | | | | | | | |
| **Contributing Factors in Resolving Family Health Crises** | The Individual Role of the Family Caregiver in Health Engagement |  | * | * | * | * |  | * | * | * | * | * | * |
|  | The Role of Professionals in to Health Engagement | * | * | * |  | * | * | * | * | * | * |  | * |
|  | The Role of Interpersonal Factors in Health Engagement |  | * |  |  | * | * | * | * | * |  | * | * |
|  | | | | | | | | | | | | | |
